# *Helicobacter pylori* colonization in Nepal; assessment of prevalence and potential risk factors in a hospital-based patient cohort

**DOI:** 10.1186/s13104-016-1867-z

**Published:** 2016-02-02

**Authors:** Shamshul Ansari, Rajendra Gautam, Hari Prasad Nepal, Shankar Nand Subedi, Sony Shrestha, Fuleshwar Mandal, Brihaspati Rimal, Muni Raj Chhetri

**Affiliations:** Department of Microbiology, Chitwan Medical College, Bharatpur, Chitwan Nepal; School of Public Health and Community Medicine, Chitwan Medical College, Bharatpur, Chitwan Nepal; Department of Biochemistry, Chitwan Medical College, Bharatpur, Chitwan Nepal

**Keywords:** Colonization, HpSAg, *Helicobactor pylori*, Nepal, Prevalence, Risk factors

## Abstract

**Background:**

*Helicobacter pylori*, a gram-negative bacterium, can cause gastritis, peptic and duodenal ulcers. It is considered an important public health problem for both developed and developing world. This bacterium is classified as the class 1 carcinogen because it can cause cancer.

**Methods:**

A hospital based study was conducted at Chitwan Medical College Teaching Hospital (CMCTH) from May to October 2014. Stool samples were collected from the suspected patients and were subjected to detection of the *H. pylori* stool antigen (HpSAg) following the procedures recommended by the manufacturer. A standard questionnaire on the potential risk factors was also designed and completed.

**Result:**

HpSAg was detected in 16 % of suspected patients. The children up to 10 years of age were found to be highly infected (36 %). The patients living in urban area were found more susceptible to develop *H. pylori* infection (P < 0.05). Tea drinking and repeated eating habit (more than twice a day) were listed as the important factors that can limit the *H. pylori* infections significantly (P < 0.05).

**Conclusion:**

In this hospital based study, a significant rate of prevalence was evaluated. However, we recommend a community based extensive study to reveal the real scenario of *H. pylori* infection in Nepalese populations.

## Background

*Helicobacter pylori* (*H. pylori*), established in 1982 by Robin Warren and Barry Marshall as the causative agent of gastritis and peptic ulcer, is a spiral, Gram-negative, micro-aerophilic bacterium [1, 2]. Before the discovery of Warren and Marshall, the human stomach was believed to be a sterile area but now, *H. pylori* is recognized as the most common cause of gastritis, which can lead to the development of more severe gastrointestinal complications such as peptic and duodenal ulcers. Because of its causal relationship to gastric adeno-carcinoma, one of the world’s deadliest cancers, the organism is classified as a class 1 carcinogen [3, 4]. Due to its capability to form biofilm and to convert from spiral to a possibly viable but non-cultivable coccoid form, the survival can be favored which can play the major factors in the epidemiology of the bacterium [5, 6].

*H. pylori* resides in upper gastrointestinal tract of more than 50 % of the world’s population. The incidence of infection is higher in developing countries and decreasing in western countries. *H. pylori* is helical shape and is thought to have evolved to penetrate the mucous lining of the stomach [7, 8]. The vast majority of infections are asymptomatic but infected individuals often have histological evidence of gastritis [9]. Related gastro-duodenal disorders can be developed in approximately 20 % of persons infected with *H. pylori* during their lifetime [10]. Although, the *H. pylori* is a public health problem in both developed and developing countries [8], its annual incidence is more in developing countries in comparison to developed countries [11]. About 50–70 % of *H. pylori* strains in Western countries carry the genes of *cag* pathogenicity island (*cag* PAI) which can increase its pathogenicity [12]. Type IV secretion system expressed by the *cag* PAI after attachment of *H. pylori* to stomach epithelial cells, injects the inflammation-inducing agent (peptidoglycan) and *cag* PAI-encoded protein CagA from their own cell wall into the epithelial cells [13] where it disrupts the cytoskeleton, adherence to adjacent cells, intracellular signaling, cell polarity, and other cellular activities [14].

Childhood and early adult years appear to be the greatest risk age for development of infection [15]. Documented risk factors also include low socioeconomic status, overcrowding, poor sanitation or hygiene, and living in a developing country [8]. Therefore, this study was aimed to document the prevalence of *H. pylori* colonization and its risk factors in suspected Nepalese population.

## Methods

### Study design and area

A hospital based study was conducted from May to October 2014 at Chitwan Medical College Teaching Hospital (CMCTH), a 600 bed tertiary care center in the city of Bharatpur, Chitwan district of central Nepal.

### Study population

A total of 100 patients with the clinical features of gastritis and other gastric abnormalities visiting out-patient department during 6 months of study period were enrolled in this study.

### Ethical aspects

All the participants were explained about *H. pylori* infection, pathogenicity, risk factors and importance of the study in local language. Both the verbal and written informed consents were taken from each of the participants (or their parents). This study was approved by the Institutional Review Committee (IRC) of CMCTH, Bharatpur, Chitwan, Nepal.

### Data collection

After obtaining consents, the patients were requested to complete the questionnaire on potential risk factors which included (*a*) *basic socio-demographic characteristics* (age, sex, ethnicity, number of family members, family education home area); (*b*) *habitual factors* (tea drinking habit, level of oral hygiene, type and frequency of food consumption); and (*c*) *any present medical history* (gastritis and any gastric abnormality). In addition, instructions were given to the patients for collection of appropriate stool samples.

### Exclusion criteria

Patients taking any antimicrobials, proton pump inhibitors or bismuth compounds within 2 weeks were excluded in this study.

### Sample collection

Sufficient quantity (1–2 ml or 1–2 g) of stool samples were collected in the morning in a clean and dry specimen collection container to obtain maximum antigens if present in the sample. Care was taken to ensure that the samples were not contaminated with urine. To obtain the best results, the test assay was performed within 6 h of sample collection. In case, when processing was not possible within 6 h, the stool samples were stored at 2–8 °C for 3 days.

### Laboratory processing

*H. pylori* antigen present in stool specimen (HpSAg) was detected by immuno-chromatographic method according to the manufacturer’s instruction. In briefly, for solid specimens, the specimen collection applicator was stabbed randomly into the fecal specimen in at least three different sites to collect approximately 50 mg of feces and then applicator was placed in the specimen collection tube containing the dilution buffer. For liquid specimens, the fecal specimen was aspirated and then two drops of sample was transferred into the specimen collection tube containing the dilution buffer. The screw cap of collection tube was tighten and mixed properly. The mixed preparation was left for 2 min to prepare the sample solution. Two drops of the sample solution was put in the sample well of the cassette. Test result was recorded in 10 min.

### Interpretation of test result

Appearance of colored line in the test line region (T) in addition to the colored line in control line region (C) was interpreted as positive result and appearance of colored line only in the control line region (C) and not in test line region (T) was interpreted as negative result.

### Performance of the method

*H. pylori* stool antigen (HpSAg) has been shown to have over 95 % correlation with the reference methods for diagnosing *H. pylori* infection. HpSAg has >95 % sensitivity, >94 % specificity, and correlation to endoscopy of 95.5 % as claimed by manufacturer. HpSAg is also non-invasive and costs a fraction of what is usually charged for endoscopy.

### Statistical analysis

Statistical analysis was performed using SPSS-16 version. Association of *H. pylori* colonization with socio-demographic factors, habitual factors and present medical history were assessed by using Chi square test. P < 0.05 were considered statistically significant.

## Results

### Frequency and distribution

Among a total of 100 patients (60 % males and 40 % females) enrolled in this study, overall prevalence of colonization of *H. pylori* was found to be 16 % (male-13.3 % and female-20.0 %). Highest number (36.0 %) of the patients was under the age of 10 years followed by 28.0 % from 11 to 20 years and 8.0 % above 60 years. Out of 16 positive cases, 75.0 % were below 10 years and remaining 25.0 % in the age of 21–40 years (Table 1).

**Table 1:** Age and gender wise distribution of total and positive cases

Age group	Total cases (n = 100)		Positive cases (n = 16)	
	Male no.	Female no.	Male no. (%)	Female no. (%)
Up to 10 years	20	16	4	8
11–20 years	20	8	0	0
21–40 years	12	4	4	0
41–60 years	4	8	0	0
More than 60 years	4	4	0	0
Total	60	40	8 (50)	8 (50)

### Socio-demographic risk factors and *H. pylori* colonization

Among the several socio-demographic risk factors assessed in this study, the age up to 20 years, females by gender, people with large family (5–10 members) and education up to primary level were found to have contributions in higher rate of colonization but the association was non-significant (P > 0.05). The urban home area contributed significantly in *H. pylori* colonization compared to rural home area (P = 0.007) (Table 2).

**Table 2:** Socio-demographic risk factors

Variable	Total cases no = 100	Positive cases no = 16 (%)	χ^2^ values	P values
Age			1.0003	0.317
Up to 20 years	64	12 (18.7)		
Above 20 years	36	4 (11.1)		
Gender			0.7937	0.372
Male	60	8 (13.3)		
Female	40	8 (20.0)		
Racial ethnicity			0.0104	0.918
Aryan	76	12 (15.8)		
Others	24	4 (16.7)		
No. of family members			2.79	0.095
Up to 4	44	4 (9.0)		
5–10	56	12 (21.4)		
Family education			1.785	0.181
Up to primary	60	12 (20.0)		
Above primary	40	4 (10.3)		
Home area			7.059	*0.007*
Urban	24	8 (33.3)		
Rural	76	8 (10.5)		

### Habitual risk factors and *H. pylori* colonization

Significant association (P = 0.0078) of *H. pylori* colonization was found in peoples with no tea drinking habit (33.3 %) and in those consuming meals only for two times a day (33.3 %) (Table 3).

**Table 3:** Habitual risk factors

Variable	Total cases no = 100	Positive cases no = 16 (%)	χ^2^ values	P values
Tea drinking habit			*7.05*	*0.0078*
Yes	76	8 (10.5)		
No	24	8 (33.3)		
Oral hygiene/visiting dentist at least once a year			0.085	0.770
Yes	28	4 (14.3)		
No	72	12 (16.7)		
Type of food consumption			3.048	0.080
Vegetarian	12	4 (33.3)		
Non-vegetarian	88	12 (13.6)		
Frequency of meal consumption per day			*7.05*	*0.0078*
Two times	24	8 (33.3)		
More than two times	76	8 (10.52)		

### Present medical history and *H. pylori* colonization

Statistically non-significant association (P > 0.05) was found between *H. pylori* colonization with potential risk factors like gastritis (16.7 %) and other gastric abnormalities (11.8 %) (Table 4).

**Table 4:** Present medical history

Variable	Total cases no = 100	Positive cases no = 16 (%)	χ^2^ values	P values
History of gastritis			0.085	0.770
Yes	72	12 (16.7)		
No	28	4 (14.3)		
Any other gastric abnormalities			2.836	0.092
Yes	68	8 (11.8)		
No	32	8 (25.0)		

## Discussion

The methods based on the isolation of bacteria are considered as the gold standard method for diagnosis of *H. pylori* infection. Because of the restriction of invasive procedures in patients such as pediatric group, the detection of bacteria revealed by stool antigen test is gaining popularity as it is less expensive, more patient-friendly than invasive testing and it is the accurate noninvasive diagnostic test that avoid the use of endoscopy in large groups of patients with dyspeptic symptoms, thus the use of noninvasive testing for *H. pylori* is being strongly recommended [16, 17]. Furthermore, in the case of monitoring the result of the eradicating therapy and for those patients who did not demand the endoscopic diagnosis, the noninvasive tests are recommended as the first diagnostic option [16]. HpSAg is a sensitive and specific noninvasive test in the diagnosis of *H. pylori* infection and it is also inexpensive as well as easy to perform with high accuracy in patients untreated for *H. pylori* infection. HpSAg has also shown promising results in adults for the non-invasive diagnosis of gastric infection [18]. However, the method has some drawbacks as the patients may be reluctant to collect stool specimens and the refrigerated stools are more difficult to perform for HpSAg detection.

The varying rate of prevalence, ranging from 5 to 60 %, of *H. pylori* infection has been documented by several authors [19–23]. In this study, we documented the prevalence of 16 % which is in accordance with the reported results of 14.2 % by Portorreal et al. from Brazil [24] and 23.6 % by Ceylan et al. from Turkey [25]. As much as 42.7 % of colonization rate was reported by Rafeey et al. from Iran [26]. The higher colonization rate may be because of selection criteria of patients in which only children were selected and it was found that the children were more colonized than other age group of patients. Early childhood is observed to be associated with *H. pylori* infection, which is acquired almost always within the first 5 years of life [27] and the colonization rate is higher in children of developing and poor countries in particular than in children of developed countries. The environmental factors of children, such as education level of parents, number of siblings, and economic factors play an important role in *H. pylori*-associated infections [28–30]. Nepal is a developing country and we observed the presence of HpSAg in 75.0 % of individuals below 10 years of age and remaining 25.0 % in the age group of 21–40 years. Similar prevalence (84 %) was also reported in children aged 6–9 years by Mahalanabis et al. from Bangladesh, the other developing country [22].

As reported in most other studies [31, 32], we also found that females were at higher risk for the colonization of *H. pylori* than males but the association was not significant. The hormonal differences between the two genders have been speculated as the explanatory description for this higher risk in females than males at this moment [33]. Marker of socioeconomic status, particularly education level has been considered as one of the important determinants of *H. pylori* prevalence in both developed [29] and developing countries [34]. We also found that the large number of family members and lower education level contributed for the colonization but the association was not significant (P > 0.05). Similarly, Rosenstock et al. from Denmark claimed that the short duration of schooling beside low socioeconomic status increases the likelihood of *H. pylori* infection [29] and similar report of lower education level in colonization of *H. pylori* was also found by Talaiezadeh et al. from Iran [35].

Nowadays, the rapid change has contributed unprecedented population growth, accompanied by rapid and unplanned urbanization resulting in large increase in urban slums without proper management of water and wastes. The peoples, living in urban slums are migrants from rural areas; lack the immunity to urban diseases posing an excellent environment for communicable diseases to spread [36]. A significantly higher rate of infection in an urban population (78.8 %) than in a rural population (69.2 %), have been reported by Kawasaki et al. from Nepal and Hoang et al. from Viet Nam [37, 38]. Likewise, 25.8 % of urban children infected with *H. pylori* were also reported by Ceylan et al. from Turkey [25].

Among several habitual risk factors assessed in our study, consumption of tea was found to significantly limit the colonization of *H. pylori* (P = 0.0078). Nowadays, tea is used as the most popular beverage in the world and its consumption has been reported to lower the rate of *H. pylori* colonization in vivo and in vitro by several authors [39–41]. Tea extracts such as catechins inhibit the growth of several pathogenic bacteria like *Staphylococcus aureus, Staphylococcus epidermidis, Vibrio* spp., *Campylobacter jejuni* and *Plesiomonas shigelloides* in vitro [42] and have killing activity to meticillin-resistant *Staphylococcus aureus* (MRSA) in vitro [43]. The tea catechins like epigallocatechin gallate, gallocatechin gallate, gallocatechin, and epigallocatechin were found to strongly inhibit urease activity, which leads to the prevention of *H. pylori* infection [40].

A number of noxious agents provided by diet can contribute synergistically in *H. pylori* pathogenicity or diet can act as protective agents [44, 45]. In current study, the meal consumption for more than twice a day significantly limited the *H. pylori* colonization (P = 0.0078). Strict vegetarian diet was observed as the insignificant contributing factor for *H. pylori* infection as one-third (33.3 %) of strict vegetarians were found to be colonized in this study. Interestingly, more or less equal rate of colonization was observed in peoples who took care of oral hygiene than those who did not.

When the bacterium causes symptoms, they usually have either symptoms of gastritis or peptic ulcer disease but in our study, the gastritis was not found as an aggravating factor as nearly equal rate of *H. pylori* colonization was observed in patients with gastritis and without gastritis depicting that the development of gastritis can be multi-factorial. However, gastric abnormalities other than gastritis was observed as non-significant (P = 0.092) enhancing factors for *H. pylori* colonization in this study.

## Conclusion

Since our hospital based study indicate 16 % prevalence rate of *H. pylori* colonization in Nepalese population, we recommend an extensive and community based study to reveal the exact scenario of this infection. Of the various risk factors evaluated to contribute in colonization of *H. pylori* in this study the patients who live in urban area, have no tea drinking habit and have lesser frequency of meals, were found to be more susceptible to harbor the organism. We also conclude that the data of this study will be supportive for extensive study on community level in the future as this report is one of the rare studies conducted so far on *H. pylori* in Nepal.

## Abbreviations

CMCTH: Chitwan medical college teaching hospital
*H. pylori*: *Helicobacter pylori*
HpSAg: *Helicobacter pylori* stool antigen
